# How personality traits and cultural value orientation moderate the pathway from musical emotional expression to group cohesion in choral rehearsal

**DOI:** 10.3389/fpsyg.2026.1905573

**Published:** 2026-10-21

**Authors:** Shixue Dong, Rui Li

**Affiliations:** College of Education, Binzhou Vocational and Technical University, Binzhou, Shandong, China

**Keywords:** behavioral synchrony, choral rehearsal, cultural value orientation, emotional expression, group cohesion, personality traits

## Abstract

**Introduction:**

Choral singing is widely valued for building cohesion in community, educational, and well-being settings, yet the route by which emotional expression during rehearsal becomes group cohesion remains poorly specified, and most work still centers on individual feeling rather than on group-level interaction. Grounded in emotional contagion and social identity theory, this study proposes and tests a dual-moderated mediation model.

**Methods:**

We surveyed 612 singers nested within 38 amateur choirs at three separated points in time and coded behavioral synchrony from audio- and video-recorded rehearsals, sampling choirs in two Chinese regions chosen to maximize variation in individualism–collectivism rather than to represent two national cultures.

**Results:**

Multilevel structural equation modeling supported a serial pathway: musical emotional expression raised group cohesion through behavioral synchrony and then shared affect, with Monte Carlo confidence intervals confirming the chain and with reversed-order and parallel-mediator models fitting less well. Extraversion and agreeableness steepened the front segment from expression to synchrony, whereas cultural value orientation, measured in each singer rather than assigned by sampling site, conditioned the rear segment from shared affect to cohesion, the conversion being stronger under a collectivistic orientation. Because 38 ensembles buy limited precision at the choir level, a Monte Carlo power analysis accompanies the estimates and marks which of them rest on firmer ground.

**Discussion:**

By giving the expression-cohesion transition an observable behavioral stage, and by locating personality and cultural moderators on distinct path segments, the study joins contagion and identity accounts. Because the design separates measurements in time but manipulates nothing, the implications for choral education and group facilitation are offered as testable guidance rather than as settled practice.

## 1 Introduction

Choral singing occupies an unusual place among collective art forms: bodies must breathe together, voices must align in pitch and timing, and personal expression must bend toward a shared sonic outcome. This blend of physiological coordination and esthetic cooperation has made amateur and community choirs an attractive setting for social and psychological intervention (Clift and Hancox, 2001). Community development programs report that ensemble participation broadens social networks and strengthens belonging among otherwise loosely connected residents (Bailey and Davidson, 2005). School and university choirs serve musical training and, at the same time, cultivate cooperation, discipline, and peer attachment (Hallam, 2010). Group singing has also entered interventions for loneliness, depressive symptoms, and social withdrawal, with several trials reporting gains in subjective wellbeing that outlast the rehearsal period (Kreutz et al., 2004). One dependent variable recurs across these literatures, sometimes named and sometimes merely implied: group cohesion. If cohesion is the product through which choral activity delivers its wider social benefits, then how rehearsal generates it becomes a question of both theoretical and practical weight.

Research on the affective dimension of music has expanded considerably, yet it remains oddly individual-centered. Much of the work on musical emotion examines how a single listener or performer perceives, feels, and physiologically responds to expressive cues (Juslin and Västfjäll, 2008). Studies of group dynamics, meanwhile, have mapped how shared activity, common goals, and interaction frequency build cohesion in teams and small groups (Beal et al., 2003), and a parallel strand documents emotional contagion—the tendency for affect to spread between people through mimicry and feedback (Hatfield et al., 1993). The trouble is that these threads rarely meet. We still know relatively little about the mechanism by which emotional expression enacted at the group level during rehearsal is converted into felt cohesion; the mediating pathway between collective affect and collective bonding has seldom been modeled directly (Barsade, 2002). Where mediation has been proposed, it is often inferred from cross-sectional correlations rather than tested against the moment-to-moment interaction that rehearsal actually involves.

A further gap concerns who is singing and where. Personality plausibly shapes how strongly a person catches and transmits emotional cues, yet traits are rarely modeled as moderators of the expression-cohesion link (Costa and McCrae, 1992). Cultural value orientation adds a second layer of variation: the same expressive intensity may carry different social meaning, and produce different bonding outcomes, in collectivistic versus individualistic settings (Hofstede, 1991). Cultural position, however, is usually inferred from where a sample happened to be collected rather than measured in the people who compose it, which leaves regional labels doing analytic work they cannot support. Three further limitations recur: samples seldom span more than one cultural setting, affect is measured statically rather than as an unfolding process, and the nested structure of choir data, with singers inside sections inside ensembles, is rarely handled through multilevel modeling (Raudenbush and Bryk, 2002). None of these is a mere inconvenience; together they obscure the conditions under which expression actually translates into cohesion.

This motivates the present study. Drawing on emotional contagion theory and social identity theory, we treat musical emotional expression as the rehearsal-level input, group cohesion as the outcome, and the spread of affect as the route between them. Onto that mediated pathway we graft two moderators acting at different points: personality as an individual-level conditioner of uptake, and cultural value orientation as a value-based conditioner of interpretation.

Three contributions follow, and it is worth stating their order of weight. First, the transition from expression to cohesion acquires an observable behavioral stage, so the mechanism can be coded from what singers do rather than inferred from what they later report. Second, the two moderators sit on distinct segments of the chain instead of entering as an undifferentiated block, which lets contagion and identity accounts be tested where each is most plausible. Third, the nested structure of rehearsal data is modeled explicitly. Because a serial chain is only one of several defensible orderings, we also state the competing accounts and test them against the data rather than assume the order we prefer.

## 2 Theoretical foundation and research hypotheses

### 2.1 The relationship between musical emotional expression and group cohesion

Within the rehearsal setting, musical emotional expression refers to the affective signals that singers generate and exchange while interpreting a work together, through dynamics, phrasing, facial display, and the subtler adjustments of vocal timbre. Three dimensions, too often blurred, are worth separating. The first is emotional arousal, the intensity of affect activated by the music itself (Eerola and Vuoskoski, 2011). The second is emotional congruence, the degree to which members converge on a shared affective reading of a passage rather than pulling in different directions (Gabrielsson, 2001). The third is expressive alignment, the perceived temporal fit of expressive behavior, where dynamics swell and recede in concert and breathing settles into a common rhythm (Muller and Lindenberger, 2011). We use expressive alignment, rather than the looser term emotional synchrony, because synchrony is reserved throughout this article for the observer-coded mediator introduced in Section 2.2. The three dimensions are related but separable: a section can be highly aroused yet incongruent, or congruent in feeling yet ragged in execution.

Since the constructs in the model lie close to one another, their boundaries deserve to be fixed before any mediation chain is proposed. Expressive alignment belongs to the independent variable and is judged by singers themselves about the sound and feel of a passage. Behavioral synchrony, in contrast, is a property of the observable record: trained raters who never see the questionnaires score how far respiratory cycles and rhythmic onsets coincide across members. Shared affect is experiential and collective, indexed by convergence among members’ concurrent emotion ratings rather than by any one member’s intensity. Group cohesion, finally, is a durable evaluation of the ensemble as a unit, commitment to its task and attraction to its members, rather than a momentary state during a single passage. The four constructs therefore differ in level, in data source, and in time frame, and Table 1 records those differences alongside the instruments used.

**Table 1:** Measurement scales, level of assessment, and reliability-validity indices for the latent constructs.

Construct	Source scale	Level and data source	Items	Scoring	Reliability/Validity
Musical emotional expression	Adapted music-affect expression inventory (Zentner et al., 2008; Juslin and Laukka, 2003)	Individual self-report, also modeled at choir level	12	7-point Likert	CR = 0.89, AVE = 0.61
Behavioral synchrony	Observer-coded respiration-onset alignment, developed here	Rater coding of rehearsal recordings	4 indices	Aligned proportion	ICC(2,k) = 0.82
Shared affect	Momentary affect convergence index (Watson et al., 1988; Bartel and Saavedra, 2000)	Within-choir convergence of concurrent member ratings	6	Dispersion-based	CR = 0.85, AVE = 0.56
Task cohesion	Group Environment Questionnaire, task facet (Brawley et al., 1987)	Individual self-report	5	7-point Likert	Cronbach α = 0.87
Social cohesion	Group Environment Questionnaire, social facet (Brawley et al., 1987)	Individual self-report	5	7-point Likert	Cronbach α = 0.84
Extraversion	Big Five Inventory, extraversion (Benet-Martinez and John, 1998)	Individual self-report	8	7-point Likert	Cronbach α = 0.83
Agreeableness	Big Five Inventory, agreeableness (Benet-Martinez and John, 1998)	Individual self-report	9	7-point Likert	Cronbach α = 0.80
Cultural value orientation	Individualism–collectivism scale (Singelis et al., 1995)	Individual self-report	16	7-point Likert	CR = 0.88, AVE = 0.58
Group cohesion (second-order)	Two-facet aggregate (Brawley et al., 1987)	Individual self-report	10	Composite	CR = 0.90, AVE = 0.64

How might such expression build cohesion? Emotional contagion theory supplies the proximal mechanism. Affect transfers between people largely without words, through mimicry of expression and posture followed by afferent feedback, so that one singer’s intensity is caught and re-radiated by neighbors (Hatfield et al., 1994). Rehearsal amplifies this loop because the channels of transfer—sound, breath, gesture—are precisely the materials of the task. Durkheim’s notion of collective effervescence extends the account to the group scale: when synchronized expressive action accumulates, it produces a heightened shared affect that members experience as something larger than themselves (Durkheim, 1995). This shared experience plausibly feeds both faces of cohesion. Coordinated affect around the musical goal strengthens task cohesion, the commitment to a common performance objective, while the warmth of jointly felt emotion strengthens social cohesion, the interpersonal attraction binding the group (Carron et al., 1985).

The empirical record, though scattered, leans in this direction. Interpersonal synchrony has been tied to greater liking and cooperation in laboratory dyads (Hove and Risen, 2009), and field studies of singing groups report that members who feel emotionally attuned also report stronger belonging (Pearce et al., 2015). Most of that evidence, however, treats emotion at the individual level and stops short of the group-level conversion at issue here. The association also runs in the other direction: ensembles that already feel close may find alignment easier, so correlation alone cannot fix the arrow, a problem we take up in Section 2.2 and again when the model is tested. Weighing theory against the available data, the balance still favors a positive link (Mullen and Copper, 1994), and we therefore advance the core hypothesis:

*H1*: Musical emotional expression during choral rehearsal positively predicts group cohesion, encompassing both its task and social components.

### 2.2 The mediating role of behavioral synchrony and shared affect

The claim in H1 is incomplete as stated, because it leaves unspecified how expression reaches cohesion. Our position is that the link is indirect, routed through what singers do with their bodies and what they then come to feel together. Social identity theory supplies the framing: cohesion grows when members categorize themselves as a single in-group, and that re-categorization feeds on salient cues of common fate and mutual coordination (Tajfel and Turner, 1979). Rehearsal is unusually rich in such cues. When a section breathes on the same upbeat and lands consonants at the same instant, every singer receives continuous evidence that the ensemble is acting as one unit (Turner et al., 1987).

Interpersonal synchrony research clarifies the first link in this chain. Coordinated movement and respiration heighten perceived entitativity and prosocial regard, an effect observed well outside music in marching, rowing, and rhythmic tapping paradigms (Wiltermuth and Heath, 2009). In singing, the relevant behavior is the joint regulation of breath and rhythm, which we treat here as breathing-rhythmic behavioral synchrony, the degree to which respiratory cycles and rhythmic onsets align across members during a passage (Vickhoff et al., 2013).

How far that alignment goes is no constant of choral singing; it depends on what is being sung. Physiological coupling is strongest when a choir sings slowly and in unison and weaker in polyphonic textures (Muller and Lindenberger, 2011; Vickhoff et al., 2013), and in one rehearsal study of amateur singers the peak frequency of heart rate variability varied considerably across members, which the authors read as evidence of limited respiratory synchronization during ordinary singing (Olsson et al., 2013). Repertoire, in short, sets a ceiling on the mediator, and our coding protocol was designed with that constraint in view. Aligned action is in any case not the endpoint. It tends to generate a felt commonality, a shared affective state in which members report experiencing the same emotion at the same moment rather than merely performing similar gestures (Valdesolo and DeSteno, 2011).

We therefore propose a sequential, two-step mediation. Emotional expression first elevates behavioral synchrony; synchrony then gives rise to shared affect; and shared affect, by furnishing the in-group cue that social identity theory requires, raises cohesion (Tarr et al., 2014). Operationally, behavioral synchrony is captured through paired respiratory and onset-timing alignment indices derived from rehearsal recordings, while shared affect is indexed by within-group convergence on momentary emotion ratings, with lower dispersion read as higher sharing (Bartel and Saavedra, 2000). The separation matters: one variable is behavioral and observable, the other experiential and self-reported, and conflating them would hide the point at which the pathway might break.

Three rival accounts deserve a hearing before the chain is asserted. Cohesion may precede the mechanism rather than follow it, since ensembles that already regard themselves as a unit may breathe together more easily and converge on feeling faster, which would turn our outcome into an antecedent. Shared affect may lead rather than trail behavior, if members who already feel alike work harder to match one another. The two mediators may also operate in parallel rather than in sequence. Theory favors the proposed order, because contagion research places mimicry and afferent feedback before the felt state and self-categorization requires a salient cue of common fate before belonging is inferred; theory alone, however, cannot settle the matter, and meta-analytic work on synchrony is itself equivocal about the ordering (Mogan et al., 2017). We accordingly estimated a reversed-order model and a parallel-mediator model as competitors, controlled for how long each ensemble had rehearsed together, and report those comparisons in Section 4.2. Accordingly:

*H2*: Behavioral synchrony and shared affect serially mediate the effect of musical emotional expression on group cohesion.

### 2.3 The moderating roles of personality traits and cultural value orientation

If the mediated pathway holds for choirs on average, it almost certainly does not hold equally for every singer or every value setting. Two sources of heterogeneity strike us as theoretically tractable, and they enter the model at different points. Personality conditions how readily a member is drawn into alignment in the first place; cultural value orientation conditions how strongly the resulting shared affect translates into commitment to the group.

Consider personality first, framed through the Big Five. Extraversion predisposes people toward stronger reward sensitivity to social stimulation and more expressive nonverbal display, so extraverted singers should both emit and absorb expressive cues more readily during rehearsal (Lucas et al., 2000). Their gain on the expression-to-synchrony link ought to be steeper than that of more reserved members, for whom the same expressive intensity registers as a weaker pull (Larsen and Diener, 1987). Agreeableness operates through a different motive, a concern for interpersonal harmony and accommodation, which inclines agreeable members to attune their behavior to the section and to suppress disruptive deviation (Graziano and Eisenberg, 1997). Where extraversion amplifies the energetic uptake of affect, agreeableness smooths the alignment of behavior, and the two need not pull together; a highly agreeable but introverted singer is an instructive boundary case, and we return to it in the discussion (John and Srivastava, 1999). Both traits, we argue, condition the front half of the chain, the segment running from emotional expression into behavioral synchrony (John et al., 2008). We treat them as relatively stable dispositions, while noting that rank-order consistency in adulthood is high rather than perfect and that agreeableness in particular can shift over a psychosocial program (Roberts and DelVecchio, 2000), a qualification resumed in Section 6. Because both traits are continuous, we probe their conditioning with simple slopes along the trait range rather than by splitting singers into types (Aiken and West, 1991).

Cultural value orientation enters downstream. Drawing on the individualism–collectivism dimension, members holding collectivistic values weight group goals above personal ones and read shared emotion as a marker of belonging worth honoring (Hofstede, 1980). For them, a given level of shared affect should convert more fully into felt cohesion, because the group’s emotional target already carries normative pull (Markus and Kitayama, 1991). Members oriented toward individualism may experience the same shared affect yet discount its bearing on their attachment to the ensemble (Triandis, 1995). Culture thus moderates the back half of the pathway, the segment from shared affect to cohesion (Oyserman et al., 2002). Because value orientation varies within any region at least as much as between regions, we measured it in each singer rather than assigning it by sampling site; region serves as a stratification variable and a covariate, never as the moderator itself. Integrating both moderators with the serial mediation of Section 2.2 yields the complete moderated mediation model, and we state the two conditional hypotheses together:

*H3*: Extraversion and agreeableness moderate the path from emotional expression to behavioral synchrony, such that the indirect effect is stronger at higher trait levels.

*H4*: Cultural value orientation moderates the path from shared affect to group cohesion, such that the conversion is stronger under collectivistic orientation, completing the proposed model.

## 3 Research model and methodological design

### 3.1 Conceptual model and variable definitions

The hypotheses developed in Section 2 assemble into a single moderated mediation framework, which Figure 1 depicts. Reading the diagram from left to right, musical emotional expression serves as the antecedent, behavioral synchrony and shared affect form the sequential mediating chain, group cohesion stands as the terminal outcome, and personality traits together with cultural value orientation enter as moderators acting on distinct segments of the chain. The figure keeps the vocabulary of Section 2.1 intact: the third dimension of the antecedent is labeled expressive alignment, since synchrony is reserved for the observer-coded mediator alone. Broken lines carry the two shorter indirect routes and the direct path, so that the schematic and the estimated model reported in Section 4.2 describe one and the same set of parameters.

**Figure 1:**
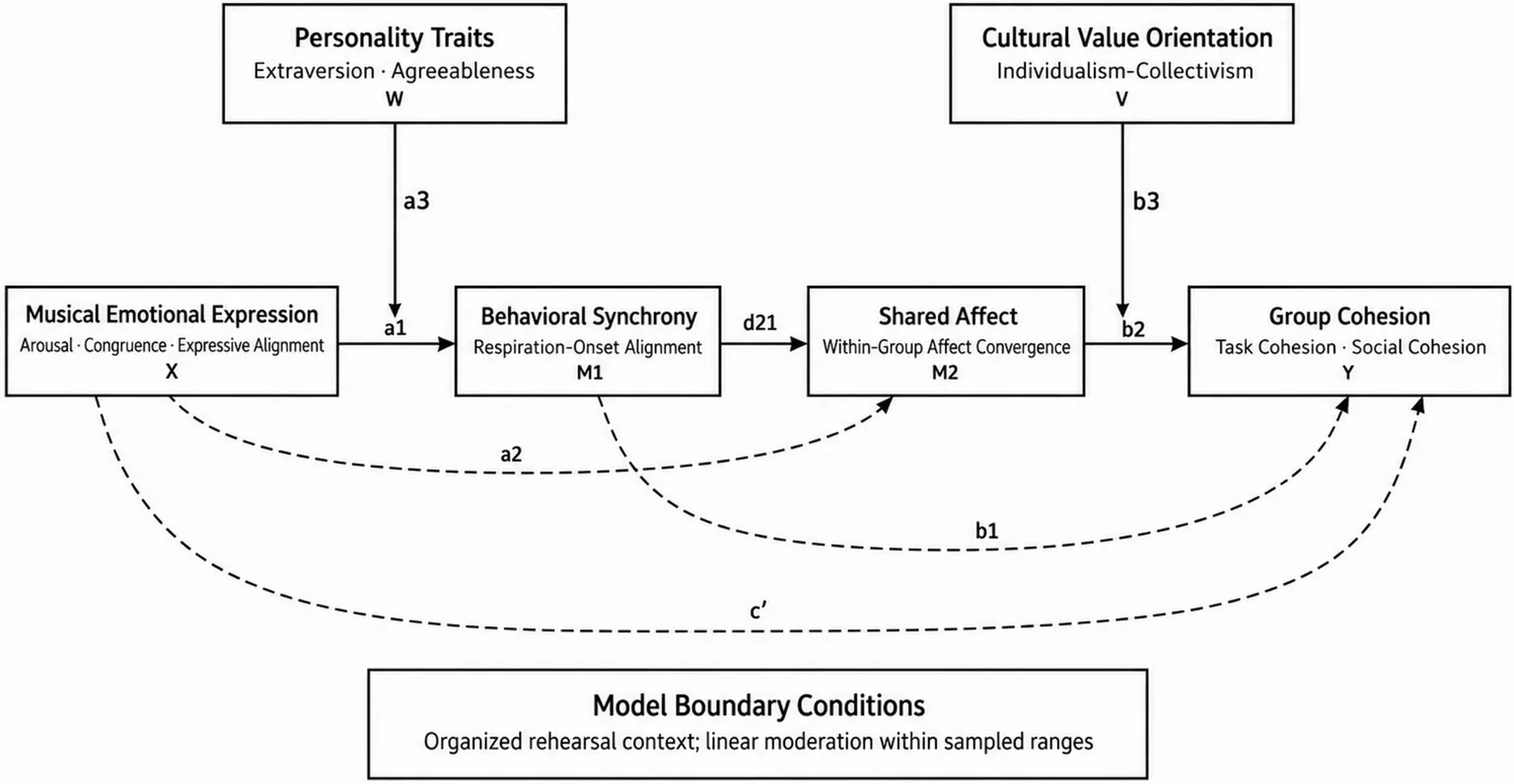
Schematic of the moderated mediation model, in which emotional expression, comprising arousal, congruence, and expressive alignment, reaches group cohesion through serial mediation by behavioral synchrony and shared affect, with personality moderating the front segment and cultural value orientation the rear segment; broken lines mark the two shorter indirect routes and the direct path.

Each latent construct is defined here before its links are specified. Musical emotional expression, the independent variable, is the affective signaling exchanged during rehearsal, with the three dimensions established earlier: arousal, congruence, and expressive alignment (Juslin and Laukka, 2003). It is reported by individual singers and also carries meaning for the ensemble as a whole, so it is modeled at both levels. Behavioral synchrony, the first mediator, denotes the temporal alignment of respiratory cycles and rhythmic onsets across members, and it is an observable behavioral construct rather than a felt one, coded by raters from recordings (Richardson et al., 2007). Shared affect, the second mediator, is the within-group convergence of momentary emotional states, operationalized inversely through dispersion among members’ concurrent ratings, and it is by definition a property of the group. Group cohesion, the dependent variable, retains its two-factor structure of task and social cohesion, following the established bidimensional conception (Brawley et al., 1987).

The two moderators differ in placement but not in level. Personality traits, scored on the extraversion and agreeableness facets of the Big Five, are individual dispositions that condition the expression-to-synchrony segment. Cultural value orientation, indexed along the individualism–collectivism continuum, is likewise measured in the individual singer and conditions the shared-affect-to-cohesion segment (Triandis and Gelfand, 1998). Neither moderator is aggregated to the region, and with only two sampling regions no inference at that level is attempted. Two boundary conditions frame the model: it applies to organized rehearsal rather than informal singing, and the moderating effects are posited as linear within the trait and value ranges sampled, an assumption revisited when the estimates are interpreted.

### 3.2 Research design and data collection procedure

Testing a chain that runs from observable behavior through felt affect to reported cohesion calls for more than a single questionnaire. We adopted a mixed design that pairs surveys administered at three separated points in time with on-site behavioral observation during rehearsal, so that the synchrony mediator could be coded from what singers actually did rather than from what they recalled doing (Creswell and Plano Clark, 2011). The design is temporally ordered but not experimental: nothing was manipulated, and the sequence of measurement supports the modeled order without establishing it. Figure 2 traces the full procedure from recruitment to the analytic dataset and carries the counts lost at each screening step.

**Figure 2:**
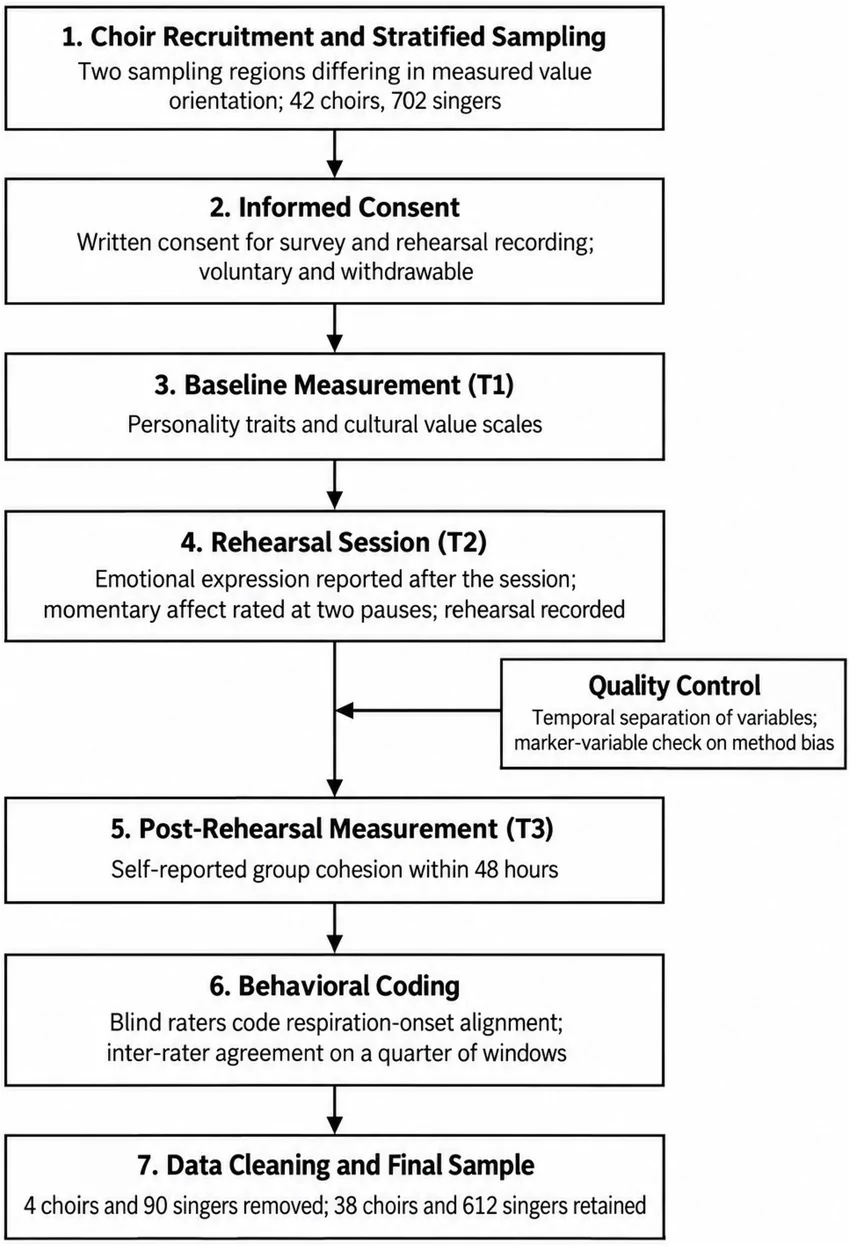
Flow of the study procedure, spanning choir recruitment and stratified sampling across two regions selected for their measured value orientation, informed consent, the three waves of survey measurement together with the momentary affect ratings taken within the recorded session, the behavioral coding, and the data-cleaning steps yielding the final analytic sample of 612 singers in 38 choirs.

All choirs were recruited in mainland China, in two regions selected because published value surveys place them at different points on the individualism–collectivism continuum, not because they belong to separate national cultures. Region A comprised three inland prefecture-level cities in Shandong Province, where amateur choirs are typically organized through work units, neighborhood committees, and retiree associations, and where membership tends to follow existing social ties. Region B comprised two coastal metropolitan cities in the Pearl River Delta, where choirs recruit by open advertisement and online platforms and draw heavily on internal migrants who arrive without prior connection to one another. Twenty choirs and 326 singers came from Region A, eighteen choirs and 286 singers from Region B. The labels collectivistic and individualistic describe the measured distribution of values in these samples; they are shorthand for a difference of degree, not a claim about two national cultures.

Sampling proceeded in strata: regions were fixed first, choirs sampled within each region across community, university, and other amateur settings, and singers nested within choirs, which preserves the multilevel structure the model requires. Because a regional contrast invites confounding, we recorded choir type, institutional affiliation, rehearsal frequency, conductor experience, choir size, and members’ age, sex, education, income bracket, and formal musical training; Section 4.1 reports the comparison.

Ethical clearance preceded every other step. The Research Ethics Committee of Binzhou Vocational and Technical University approved the protocol on 31 July 2024 under reference number BZVTU-IRB-2024-0731, and every participant signed a consent form that covered the questionnaires and the audio and video recording of rehearsal alike. All singers were adults, the youngest being eighteen, so guardian consent did not arise. Participation remained voluntary and withdrawable at any point, and withdrawal carried no consequence for a member’s standing in the ensemble (American Psychological Association, 2002).

Repertoire matters for a study that codes breathing, so it was standardized rather than left free. The choirs sang mainly Mandarin-language arrangements of Chinese folk songs and twentieth-century choral art songs, with a smaller share of Western classical, sacred, and popular arrangements. For the recorded session, every ensemble rehearsed two required pieces circulated in advance: a slow homophonic setting sung largely in unison at roughly 60 to 72 beats per minute, and a four-part polyphonic setting at a moderate tempo. Only these two pieces were coded, which keeps the synchrony index comparable across choirs and spans the textures that earlier work identifies as favorable and unfavorable to physiological coupling (Vickhoff et al., 2013; Olsson et al., 2013).

Measurement spanned three points in time, both to separate the variables and to blunt common-method bias. Trait and cultural-value scales were administered at baseline, on average fourteen days before the recorded session and never fewer than ten. Musical emotional expression was reported immediately after the recorded rehearsal at the midpoint, and momentary affect was rated on a short form at two scheduled pauses within that same session, so the self-reported mediator and the behavioral coding describe one rehearsal rather than two occasions. Cohesion was reported within 48 h afterwards. The ordering of waves follows the modeled sequence and was fixed in advance.

Behavioral synchrony was coded from synchronized audio and video. Two camcorders recorded each section from the front and the side at 50 frames per second, an ambient array captured the ensemble, and clip microphones on four to six members per choir captured breath noise; a clapperboard at the head of every take, followed by cross-correlation of the audio tracks, aligned the streams to within one frame. Respiratory cycles were identified from three converging sources: thoracic and abdominal excursion visible in the side view, audible inhalation on the clip channels, and the breath marks in the score. Rhythmic onsets were taken as phrase entries and final-consonant releases, with an alignment tolerance of 120 ms fixed after pilot coding. Recordings moved on the day of capture to an encrypted institutional server, indexed by choir and seat code rather than by name and open only to the four trained coders and the two authors, and they will be destroyed five years after publication, as the consent form specifies.

Coding was semi-automated. Four postgraduate raters annotated events in ELAN after twenty hours of training on material from choirs outside the sample, and a custom routine then converted the annotations into alignment indices over eight-second windows anchored to phrase boundaries. Every rater remained blind to the hypotheses and to all questionnaire responses. A quarter of the windows, 1,184 of 4,736, were double-coded, and agreement was estimated with a two-way random-effects, average-measures intraclass correlation, ICC(2,k) = 0.82, 95% CI [0.74, 0.88]. Each singer’s score is the proportion of coded events falling within tolerance of the section modal onset and of the ensemble modal onset; choir-level scores are the mean of member scores, an aggregation the agreement statistics support (median $r_{\mathrm{wg}}$ = 0.81, ICC(1) = 0.19, ICC(2) = 0.79) (Shrout and Fleiss, 1979; James et al., 1984).

Cleaning cost the sample less than we had feared. Forty-two choirs and 702 singers consented at recruitment; 19 singers failed one or both embedded attention checks and 31 either withdrew or missed the recorded session, while three choirs returned too few completed protocols for reliable aggregation and one choir’s side-view recording proved unusable. Removing those four choirs together with their 40 members left 38 choirs and 612 singers. Item-level missingness among the retained cases stood at 1.8 percent, showed no association with region, sex, or age, and was handled by full information maximum likelihood rather than by deletion, which avoids the bias and the loss of efficiency that listwise methods carry (Enders and Bandalos, 2001).

### 3.3 Measurement instruments and analytic strategy

Every latent construct was measured with an established scale rather than an *ad hoc* instrument, which anchors reliability and validity against prior validation work. Table 1 lists the source scale, the level at which each construct was assessed and by whom, the scoring metric, and the reliability and validity indices obtained in this sample. Responses used seven-point Likert formats throughout, the behaviorally coded synchrony index excepted, and all multi-item composites cleared the conventional thresholds, with composite reliability at or above 0.70 and average variance extracted above 0.50 (Fornell and Larcker, 1981).

The music-affect expression inventory deserves fuller description, since it was adapted rather than adopted wholesale. Items were drawn from the expressive-emotion descriptors of the Geneva Emotional Music Scale (Zentner et al., 2008) and from the performance literature on how feeling is encoded in vocal and musical signals (Juslin and Laukka, 2003), reworded to refer to the rehearsing group rather than to a solitary listener; the adaptation was made under the academic-use terms of the original instrument. Two bilingual translators produced the Mandarin version and a third back-translated it; a panel of four choral conductors and two music psychologists then reviewed the wording, and a pilot with 86 singers from non-participating choirs reduced fifteen candidate items to twelve, four per dimension. Sample items read: the music we were rehearsing stirred strong feeling in me (arousal); our section understood this passage in the same emotional way (congruence); and our phrasing rose and fell together (expressive alignment). The seven-point response format was applied to the Big Five scales as well, which keeps the response metric constant across instruments, and the resulting reliabilities appear in Table 1.

Because singers nest within choirs, single-level estimation would understate standard errors and conflate within- with between-choir variance. We therefore specified a multilevel structural equation model in Mplus 8.10, estimated by robust maximum likelihood, partitioning each variable into its within- and between-choir components and testing the serial mediation at the level where the theory locates it (Preacher et al., 2010). Musical emotional expression and behavioral synchrony carry meaning at both levels and were modeled accordingly. The two moderators are individual-level variables, group-mean centered so that their interactions are estimated strictly within choirs and are not contaminated by between-choir composition (Enders and Tofighi, 2007); region entered as a between-choir covariate rather than as a moderator, since with 38 ensembles and two regions no inference at the level of region is warranted and none is attempted. The three interaction slopes were estimated as fixed effects, because 38 clusters cannot support random slopes of that kind without straining the model. The indirect chain from emotional expression ( X ) through synchrony ( $M_{1}$ ) and shared affect ( $M_{2}$ ) to cohesion ( Y ) is the product of its constituent paths, as shown in Equation (1):

$$\mathrm{IE}=a_{1}\times d_{21}\times b_{2}$$ (1)

In this expression $a_{1}$ links X to $M_{1}$ , $d_{21}$ links $M_{1}$ to $M_{2}$ , and $b_{2}$ links $M_{2}$ to Y . The two shorter routes follow the same convention, with $a_{2}$ linking X to $M_{2}$ and $b_{1}$ linking $M_{1}$ to Y . The conditional, moderated form lets the first segment vary with personality ( W ) and the last with cultural value orientation ( V ), so the conditional indirect effect is evaluated as shown in Equation (2):

$$\mathrm{IE}(W,V)=(a_{1}+a_{3}W)\times d_{21}\times (b_{2}+b_{3}V)$$ (2)

Here $a_{3}$ and $b_{3}$ are the respective interaction coefficients. Two-level estimation and bias-corrected bootstrapping cannot be combined in the software used, so the limits reported below come from the Monte Carlo method: 20,000 draws of the relevant coefficients from their asymptotic covariance matrix, multiplied through, with the 2.5th and 97.5th percentiles of the resulting product distribution taken as the interval (Preacher and Selig, 2012). A bias-corrected bootstrap of 5,000 resamples under single-level estimation reproduced those limits to within 0.01. An indirect effect was judged reliable when its interval excluded zero, a standard that avoids the symmetry assumption normal-theory tests impose on skewed product terms (Preacher and Hayes, 2008). The competing orderings set out in Section 2.2 were estimated on the same 612 cases with the same observed variables and the same estimator, so their AIC and BIC values, computed from the robust loglikelihood with the scaling correction applied, are comparable across these non-nested alternatives; robustness checks added choir tenure, education, and rehearsal frequency as covariates. Method variance was addressed statistically as well as procedurally, through a confirmatory-factor marker variable chosen for its irrelevance to music and to group life (Williams et al., 2010), and the precision the design affords at the choir level was examined by Monte Carlo simulation (Muthén and Muthén, 2002), with both results reported in Section 4.

## 4 Data analysis and results

### 4.1 Descriptive statistics and measurement model evaluation

The final analytic sample comprised 612 singers nested within 38 choirs, 326 of them in the collectivistic-leaning region and 286 in the individualistic-leaning one. Women made up 58 percent of respondents, ages ranged from 18 to 67 (mean 34.2, SD 11.6), and choral experience averaged 6.4 years. Choir size after cleaning ran between 11 and 24 active members, which leaves enough within-choir respondents for stable aggregation at the between level. Variance decomposition confirmed that the multilevel treatment was warranted: the intraclass correlation reached 0.19 for behavioral synchrony, 0.16 for shared affect, and 0.14 for group cohesion, so between-choir differences account for a non-trivial share of the variance in every endogenous variable.

A regional contrast can easily stand in for other differences, so Table 2 sets the two subsamples side by side on the characteristics most likely to confound the comparison. Tests on member characteristics treat the 612 singers as units, whereas tests on organizational characteristics treat the 38 ensembles as units, which is why the degrees of freedom differ down the column. The regions proved similar in age, sex composition, years of choral experience, formal musical training, income bracket, choir size, and conductor experience. Two variables differed at the conventional level: singers in Region B more often held a tertiary degree, and choirs in Region A rehearsed slightly more often. Both were carried into the moderation models as covariates, and the interactions reported in Section 4.3 held with and without them. The value measure itself separated the regions sharply, which is what the sampling design intended.

**Table 2:** Comparison of the two regional subsamples on demographic, musical, and organizational characteristics.

Characteristic	Region A: collectivistic-leaning (20 choirs, 326 singers)	Region B: individualistic-leaning (18 choirs, 286 singers)	Group comparison
Age in years, M (SD)	34.8 (11.9)	33.5 (11.2)	*t*(610) = 1.39, *p* = 0.16
Women, %	59.2	56.6	Pearson $\chi^{2}$ (1) = 0.42, *p* = 0.52
Choral experience in years, M (SD)	6.6 (4.8)	6.2 (4.5)	*t*(610) = 1.07, *p* = 0.29
Formal music training in years, M (SD)	3.1 (3.4)	3.4 (3.6)	*t*(610) = −1.06, *p* = 0.29
Tertiary education, %	47.5	58.0	Pearson $\chi^{2}$ (1) = 6.72, *p* = 0.010
Income above regional median, %	48.8	51.7	Pearson $\chi^{2}$ (1) = 0.52, *p* = 0.47
University choirs, %	30.0	38.9	Pearson $\chi^{2}$ (1) = 0.33, *p* = 0.57
Rehearsals per month, M (SD)	5.9 (1.8)	5.2 (1.7)	*t*(36) = 2.14, *p* = 0.039
Active choir size, M (SD)	16.3 (3.6)	15.9 (3.4)	*t*(36) = 0.35, *p* = 0.73
Conductor experience in years, M (SD)	12.4 (6.1)	11.6 (5.8)	*t*(36) = 0.41, *p* = 0.68
Collectivistic value orientation, M (SD)	5.02 (0.96)	4.16 (1.02)	*t*(610) = 10.75, *p* < 0.001

Table 3 reports means, standard deviations, and the zero-order correlation matrix for the study variables. The pattern of associations sits comfortably with the hypothesized chain: emotional expression correlated positively with behavioral synchrony ( r = 0.46), synchrony with shared affect ( r = 0.51), and shared affect with both facets of cohesion, at 0.49 for the task facet and 0.52 for the social one, each at p < 0.01; the equally weighted composite that carries cohesion through the structural model correlated 0.54 with shared affect. Personality and cultural-value variables showed weaker direct correlations with cohesion, which is what one expects of variables that condition a relation rather than produce it (Aiken and West, 1991).

**Table 3:** Descriptive statistics and zero-order correlations among study variables.

Variable	M	SD	1	2	3	4	5	6	7
1 Emotional expression	5.12	0.84	—						
2 Behavioral synchrony	0.63	0.14	0.46	—					
3 Shared affect	4.98	0.91	0.42	0.51	—				
4 Task cohesion	5.34	0.78	0.39	0.44	0.49	—			
5 Social cohesion	5.21	0.82	0.37	0.41	0.52	0.63	—		
6 Extraversion	4.76	0.93	0.28	0.31	0.24	0.19	0.22	—	
7 Agreeableness	5.05	0.81	0.21	0.29	0.23	0.20	0.18	0.34	—
8 Cultural value orientation	4.62	1.07	0.16	0.18	0.27	0.25	0.30	0.12	0.15

Confirmatory factor analysis on the eight-factor measurement model returned an acceptable fit, $\chi^{2}$ /df = 2.31, CFI = 0.95, TLI = 0.94, RMSEA = 0.046, SRMR = 0.052. Reliability held under whichever metric suits the construct: composite reliability ran from 0.85 to 0.90 across the questionnaire scales, Cronbach’s α from 0.80 to 0.87, and the behaviorally coded synchrony index carried an ICC(2,k) of 0.82. Discriminant validity held throughout: for every construct pair the square root of the average variance extracted surpassed the inter-construct correlation, and no heterotrait-monotrait ratio rose above 0.78 (Hu and Bentler, 1999).

Two further checks address the worry that the mediators merely restate one another. Forcing behavioral synchrony and shared affect onto a single factor worsened fit appreciably ( $\Delta \chi^{2}$ = 148.6, Δ df = 7, p < 0.001; Δ CFI = 0.041), as did a model merging expressive alignment with behavioral synchrony ( $\Delta \chi^{2}$ = 121.3, Δ df = 7, p < 0.001; Δ CFI = 0.034), so the eight-factor solution was retained and the two mediators can be treated as distinct rather than as one construct under two names. Multi-group analysis across the two regions supported configural and metric invariance and then scalar invariance, with Δ CFI of 0.004 and 0.007 at the successive steps, both inside the conventional 0.01 bound (Cheung and Rensvold, 2002). The marker variable told a similarly reassuring story: it absorbed 3.1 percent of the indicator variance, left every structural path significant, and shifted no path estimate by more than 0.02, which makes shared method a poor explanation for what follows (Williams et al., 2010). The comparisons below can therefore be read as differences in the constructs themselves rather than in how the items behave.

Comparing the two regions, Figure 3 sets their construct means side by side. Expression itself scarcely differed, whereas shared affect and both facets of cohesion ran higher where collectivistic values predominate, the gap on shared affect reaching half a scale point. We report the difference rather than explain it away, but it is a difference in level and not the moderation at issue: the hypothesis concerns the rate at which shared affect converts into cohesion, which no comparison of means can settle. Section 4.3 tests that conversion directly, carrying the covariates identified in Table 2 with it.

**Figure 3:**
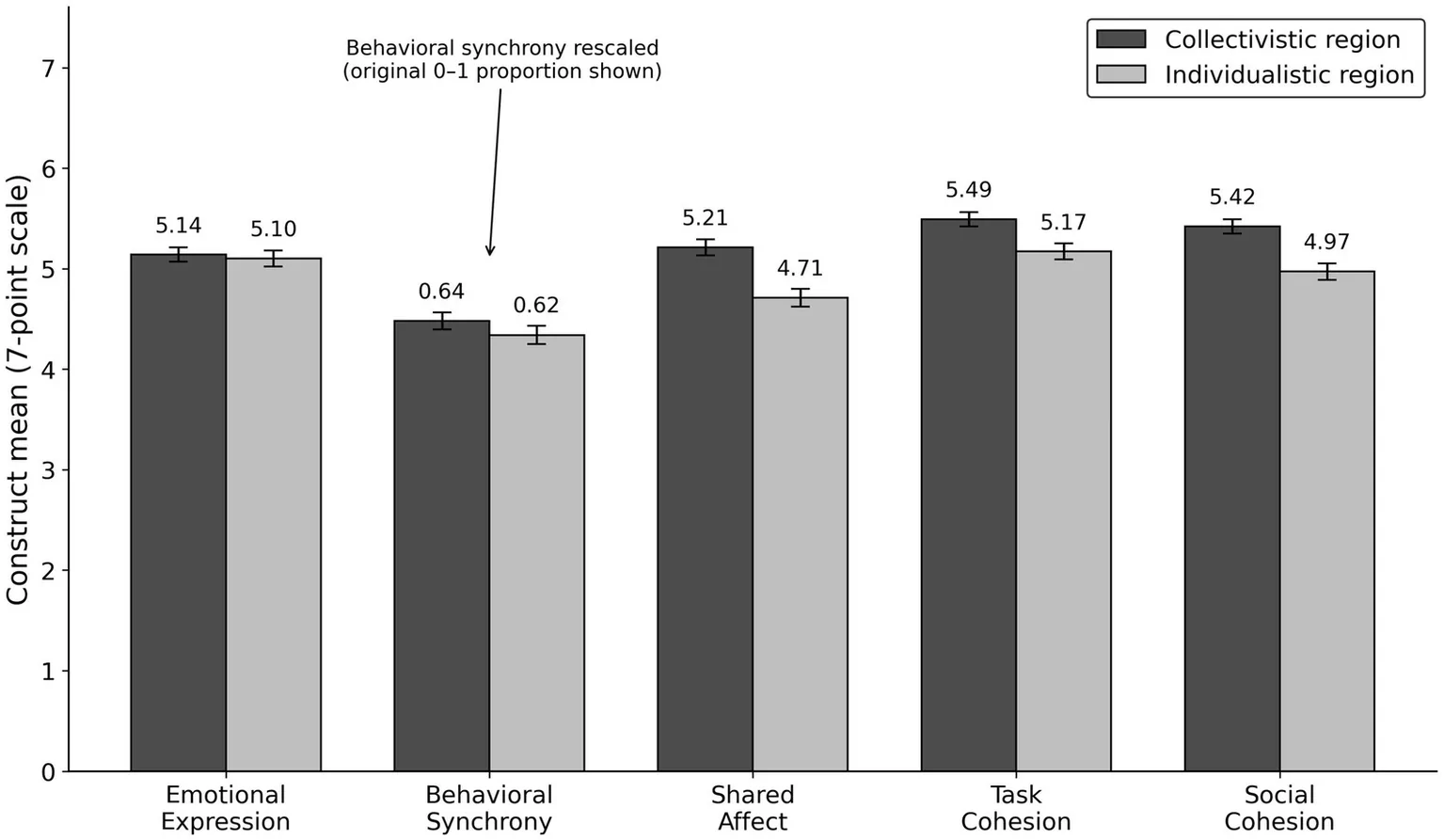
Construct means for the two sampling regions, with error bars showing standard errors and the widest gaps appearing on shared affect and cohesion; the behavioral synchrony index, a proportion bounded by zero and one, is rescaled onto the seven-point axis, and its untransformed values are printed above the bars.

### 4.2 Test of the serial mediation effect

With the measurement model in hand, we turned to the structural paths. Every link in the proposed chain reached significance and carried the expected sign. Emotional expression predicted behavioral synchrony ( $a_{1}$ = 0.43, p < 0.001), synchrony predicted shared affect ( $d_{21}$ = 0.48, p < 0.001), and shared affect predicted group cohesion ( $b_{2}$ = 0.45, p < 0.001). The two shorter routes were weaker but not negligible: expression also predicted shared affect directly ( $a_{2}$ = 0.073, p < 0.05), and synchrony predicted cohesion directly ( $b_{1}$ = 0.142, p < 0.01). The direct effect of expression on cohesion shrank yet stayed positive once the mediators entered ( $c^{\prime }$ = 0.140, p < 0.05), the signature of partial rather than full mediation (Baron and Kenny, 1986).

The rival accounts fared worse. A reversed-order model, in which shared affect precedes behavioral synchrony, fit less well than the proposed chain (AIC 24349.2 versus 24318.7; BIC 24582.9 versus 24552.4), and a parallel-mediator model fell between the two (AIC 24341.5; BIC 24566.4); all three were fitted to the same cases with the same variables and estimator, which is what makes the comparison legitimate. Adding the number of years an ensemble had rehearsed together as a choir-level covariate left the serial indirect effect essentially unchanged, at 0.089 rather than 0.093, and the same held once education and rehearsal frequency entered. None of this excludes reciprocal influence across a full season, in which cohesion feeds back into the ease of synchronizing; it does show that the proposed order describes these data better than its competitors do.

Table 4 decomposes the total effect into its direct and indirect portions, with 95% Monte Carlo confidence intervals from 20,000 draws, and lists the two shorter component paths alongside them. The full serial route, running from expression through synchrony and shared affect to cohesion, produced an indirect effect of 0.093, its interval safely clear of zero. Both single-mediator routes also held, each contributing a smaller but reliable share. Summed, the three indirect paths account for the bulk of the total effect and leave only a modest direct residual.

**Table 4:** Path coefficients and decomposition of indirect effects in the serial mediation model, with 95% Monte Carlo confidence intervals.

Effect or path	Estimate	SE	95% CI lower	95% CI upper	Proportion
Total effect	0.327	0.041	0.247	0.408	1.00
Direct effect ( $c^{\prime }$ )	0.140	0.038	0.066	0.215	0.43
Path $a_{2}$ (expression to shared affect)	0.073	0.028	0.019	0.129	-
Path $b_{1}$ (synchrony to cohesion)	0.142	0.041	0.063	0.223	-
Indirect via synchrony only	0.061	0.019	0.027	0.102	0.19
Indirect via shared affect only	0.033	0.013	0.011	0.061	0.10
Serial: synchrony then shared affect	0.093	0.024	0.051	0.144	0.28
Total indirect effect	0.187	0.033	0.124	0.254	0.57

To express how much of the transmission runs through the mediators, we computed the mediation ratio as the total indirect effect divided by the total effect, as shown in Equation (3):

$$P_{M}=\frac{IE_{\text{total}}}{c}=\frac{0.187}{0.327}\approx 0.57$$ (3)

Roughly 57 percent of the bearing of expression on cohesion therefore travels along mediated routes, and the serial chain alone carries about half of that mediated portion (MacKinnon et al., 2004). The ratio is easy to over-read. It describes rather than tests, it wanders considerably from sample to sample, and with a direct effect still significant it should be taken as a rough statement of relative weight and nothing more. Figure 4 lays the decomposition out visually, ranging the components from the direct residual to the total so that the comparative width of the intervals, and not merely the ordering of the point estimates, is legible at a glance.

**Figure 4:**
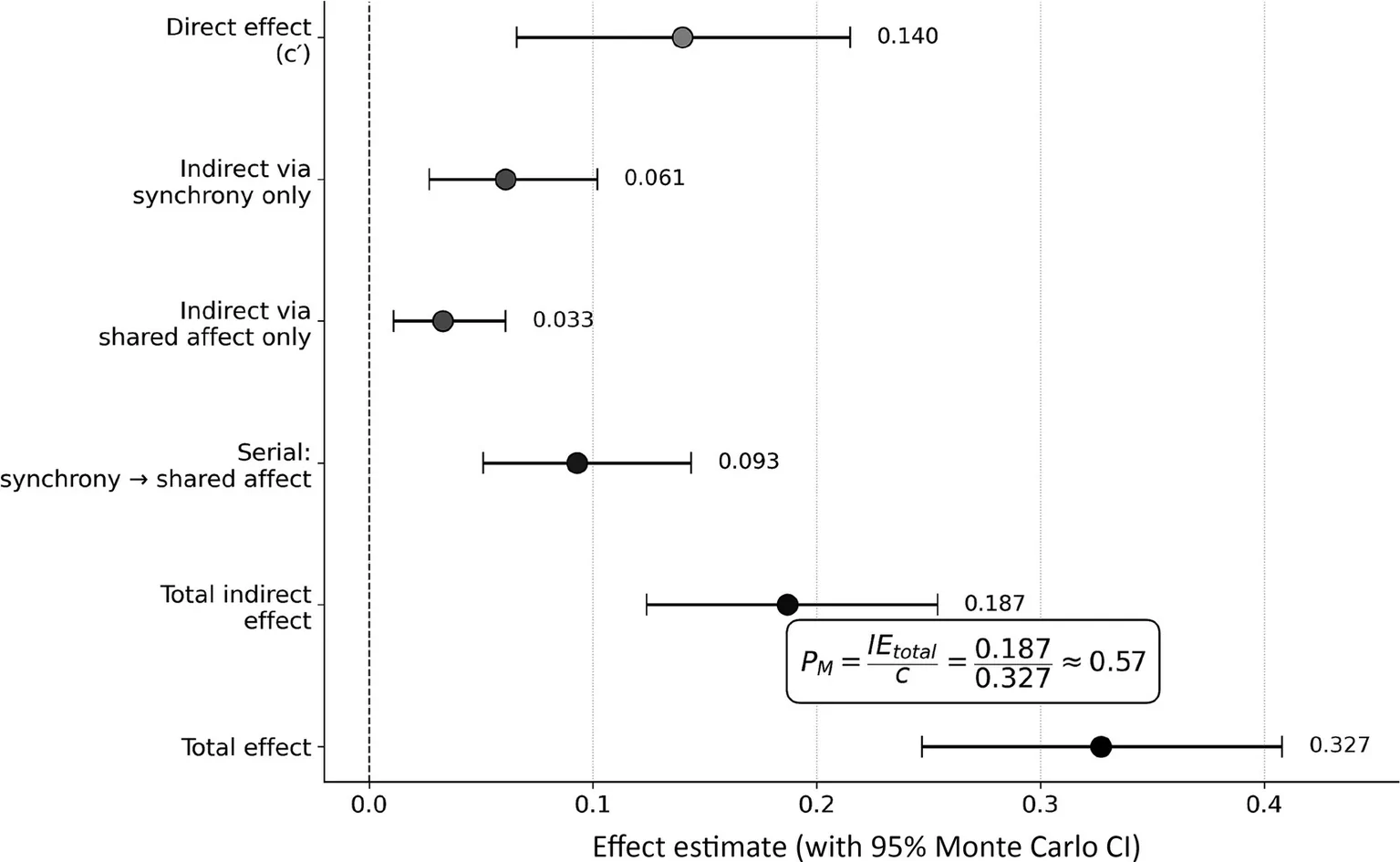
Point estimates and 95% Monte Carlo confidence intervals for the direct effect, the three indirect routes, the total indirect effect, and the total effect, with the mediation ratio shown alongside; the serial route through synchrony and shared affect is the largest of the mediated components.

These results support H2, with one qualification: the support rests on comparison against alternatives rather than on the fit of a single model. Behavioral synchrony and shared affect do carry the influence of expression onto cohesion, and they do so in sequence rather than as interchangeable parallel channels.

### 4.3 Test of the moderated mediation effects

We now ask whether the mediated chain bends with personality and culture, as H3 and H4 predict. Three interaction terms entered the model, each on the segment its parent theory implicates. The expression-by-extraversion product on the synchrony path was positive and reliable ( $a_{3}$ = 0.15, p < 0.01), and the parallel expression-by-agreeableness term held as well ( ${a_{3}}^{\prime }$ = 0.12, p < 0.05). On the rear segment, the shared-affect-by-culture product predicted cohesion ( $b_{3}$ = 0.16, p < 0.01). The moderators thus fell on the distinct segments the theory assigned them rather than scattering across the model.

Simple-slope probing made the pattern concrete. Among high-extraversion singers (+1 SD) the expression-to-synchrony slope was steep, b = 0.58, whereas among their low-extraversion counterparts (−1 SD) it flattened to b = 0.28; the corresponding slopes for agreeableness were 0.49 and 0.25. The cultural moderator behaved analogously at the other end of the chain, the shared-affect-to-cohesion link being much stronger for collectivistically oriented members ( b = 0.61) than for individualistically oriented ones ( b = 0.29). All six slopes remained positive, so the moderation is a matter of degree rather than of presence and absence. Because the predictors are group-mean centered and every variable standardized, the lines of Figure 5 pivot about a common origin: the moderators’ own positive main effects live in the intercepts and not in the plotted segments, which display conditional slopes alone (Preacher et al., 2006).

**Figure 5:**
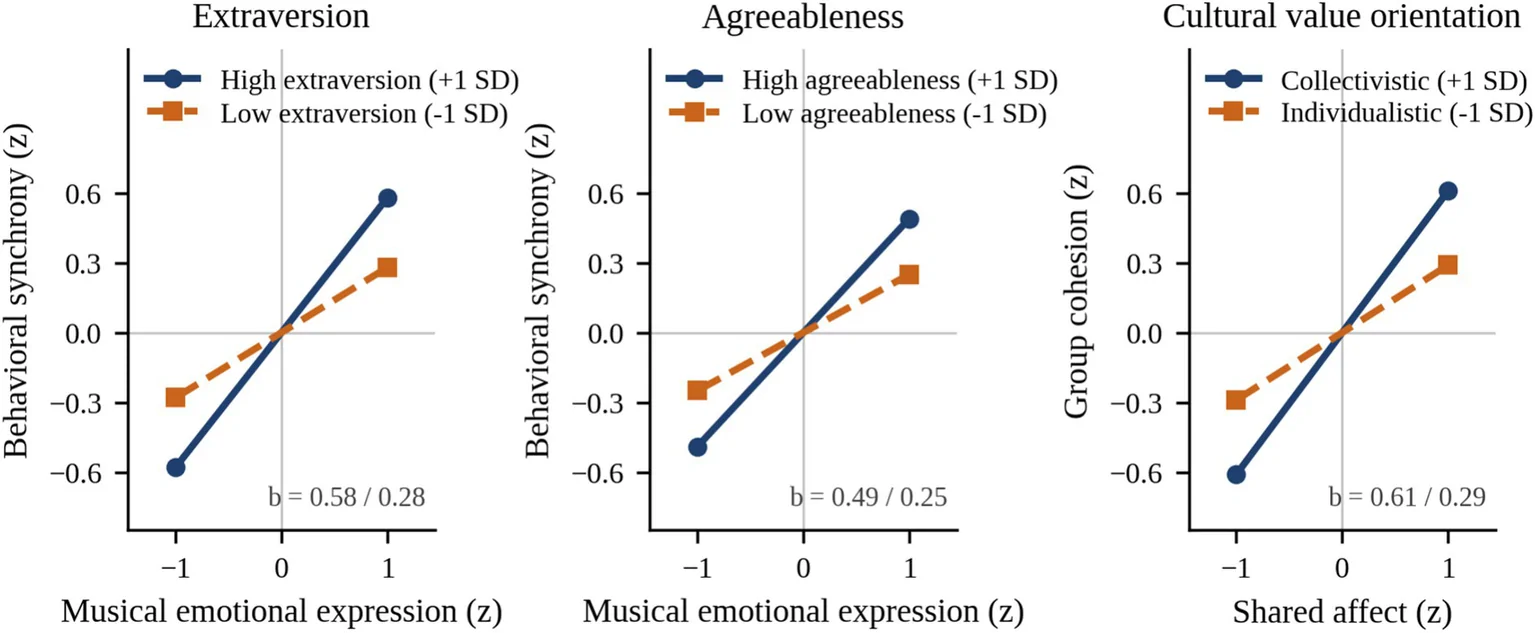
Simple slopes for the three moderated paths, showing the expression-to-synchrony slope at high and low extraversion and agreeableness and the shared-affect-to-cohesion slope at collectivistic and individualistic value orientations; all variables are standardized and group-mean centered, so the lines cross at the sample mean and the main effects of the moderators are absorbed into the intercept.

The conditional indirect effects follow from the same expression that produced the unconditional one, which is to say from evaluating Equation (2) at chosen levels of W and V rather than at their means. We evaluated it at the high and low combinations of the two moderators, taking Monte Carlo intervals as before (Preacher and Selig, 2012; Hayes, 2018).

Table 5 reports the resulting conditional indirect effects. With both moderators high the indirect effect reached 0.170; with both low it fell to 0.039, and although that interval still excluded zero, it did so narrowly. The index of moderated mediation was reliable for each of the three moderating terms taken separately, which indicates that the spread across conditions reflects moderation rather than sampling noise (Edwards and Lambert, 2007).

**Table 5:** Conditional indirect effects at high and low levels of the moderators.

Extraversion	Cultural orientation	Conditional IE	SE	95% CI lower	95% CI upper
High extraversion (+1 SD)	Collectivistic (+1 SD)	0.170	0.038	0.105	0.252
High extraversion (+1 SD)	Individualistic (−1 SD)	0.081	0.027	0.033	0.138
Low extraversion (−1 SD)	Collectivistic (+1 SD)	0.082	0.027	0.035	0.139
Low extraversion (−1 SD)	Individualistic (−1 SD)	0.039	0.016	0.013	0.075
Index of moderated mediation (extraversion)		0.032	0.013	0.010	0.059
Index of moderated mediation (agreeableness)		0.026	0.012	0.005	0.051
Index of moderated mediation (cultural value orientation)		0.033	0.013	0.010	0.061

Taken together, the mediated pathway runs strongest when an expressive, extraverted singer performs within a collectivistic value frame and weakest when neither condition obtains, which supports H3 and H4. Figure 6 summarizes the estimated model as a whole, printing the standardized coefficient on every path and distinguishing the serial chain from the two shorter routes and the direct path, so that the structure behind Table 4 can be read at a glance.

**Figure 6:**
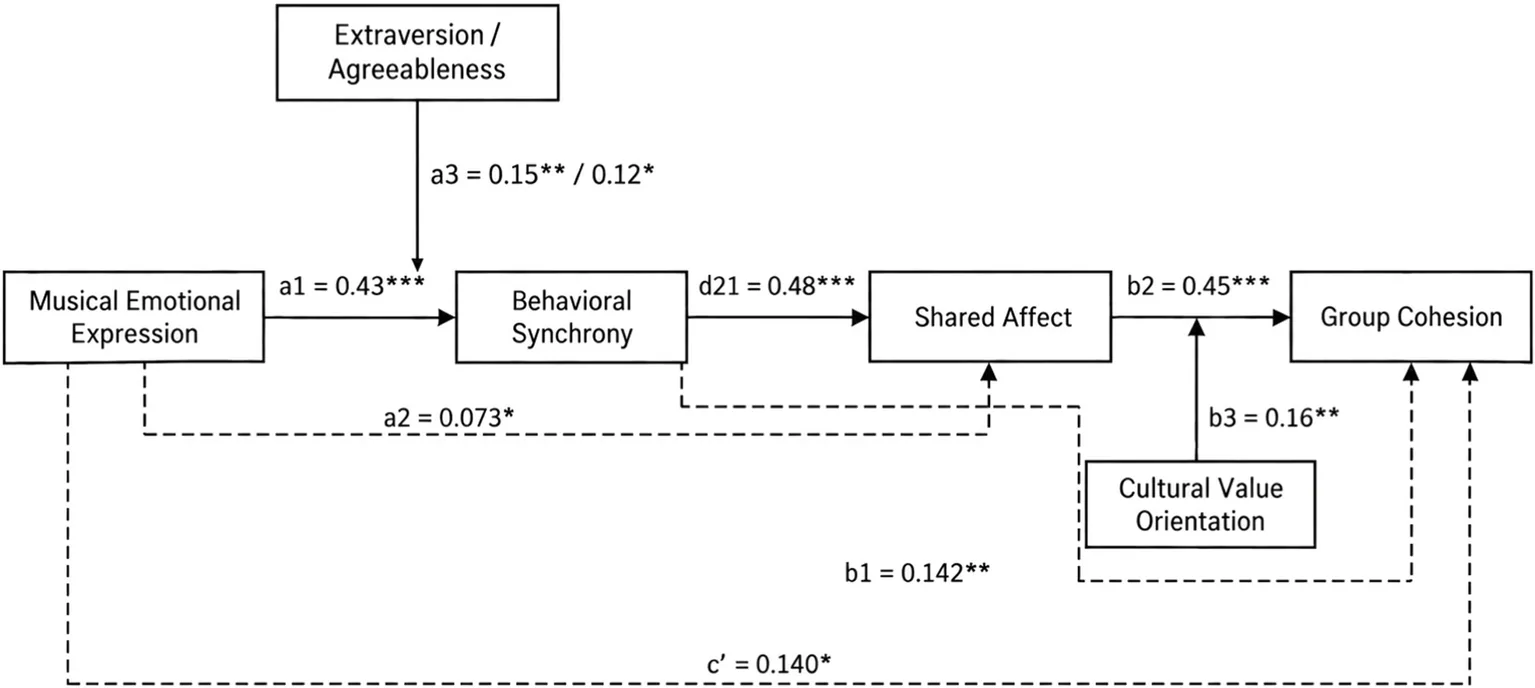
Estimated multilevel path model with standardized coefficients printed on the paths, solid lines marking the serial chain and broken lines the two shorter indirect routes and the direct path, and the two moderators placed on the segments they condition. *p < 0.05, **p < 0.01, ***p < 0.001.

Reading a moderation only at plus and minus one standard deviation hides as much as it shows, so Figure 7 traces the conditional indirect effect across the observed range of extraversion at three levels of cultural value orientation. What emerges is a gradient rather than a switch. The effect climbs steadily as extraversion rises, and the three cultural lines fan apart as they climb. The displayed 95% Monte Carlo interval is for the collectivistic line only and includes zero at the lowest extraversion values.

**Figure 7:**
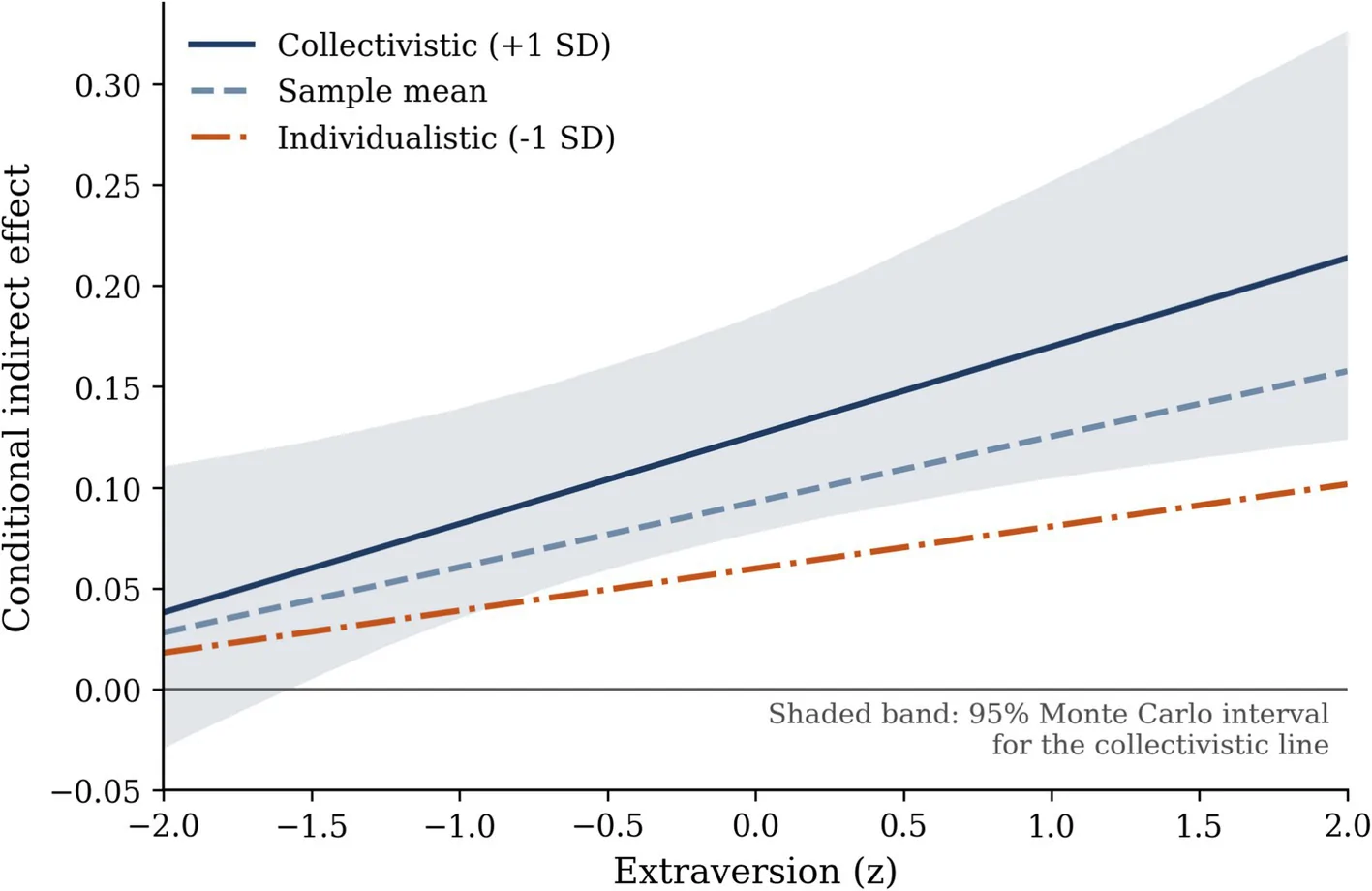
Conditional indirect effect of musical emotional expression on group cohesion through behavioral synchrony and shared affect, plotted across the observed range of extraversion at three levels of cultural value orientation, with the shaded band giving the 95% Monte Carlo interval for the collectivistic line.

Thirty-eight ensembles is a modest number of clusters, and it seemed better to quantify the resulting imprecision than to assert that it was tolerable. A Monte Carlo simulation generating 2,000 datasets from the estimated parameters, at cluster sizes matching our own, produced the power curves of Figure 8 (Muthén and Muthén, 2002). At the realized design, power to detect the index of moderated mediation stood at 0.73 for extraversion and 0.72 for cultural value orientation, but at only 0.61 for agreeableness, whose interaction is the smallest of the three and small by conventional benchmarks (Cohen, 1988). Roughly fifty choirs would carry the first two past the customary threshold and roughly sixty would carry all three, a figure worth recording for anyone designing a replication. The moderation results reported above should be read with that asymmetry in mind: two of the three indices rest on reasonable precision, the third on less.

**Figure 8:**
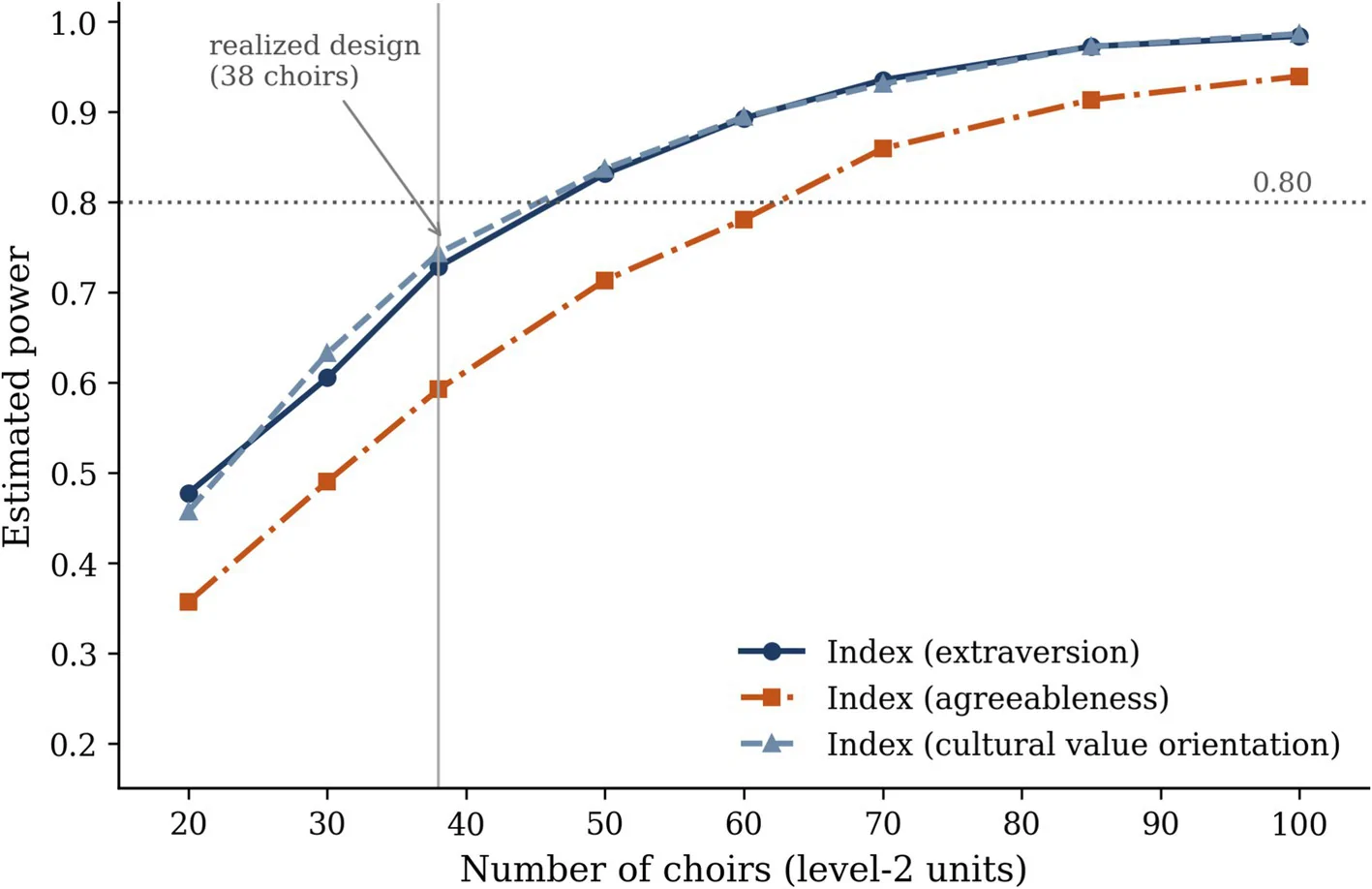
Monte Carlo estimates of statistical power for the three indices of moderated mediation as a function of the number of choirs, holding the average number of responding members per choir at sixteen; the broken line marks the conventional 0.80 criterion and the vertical line the realized design.

## 5 Discussion

The results draw a coherent picture. Emotional expression in rehearsal does not lift cohesion by some direct act of feeling; it works through what singers do and then through what they come to feel as one. The serial chain carried more of the effect than either shorter route, and the proposed order fit better than its reversed and parallel competitors, which is the evidence behind our claim that sequence matters here. Behavior comes first. When voices align in breath and rhythm, that audible coordination is what later ripens into a shared affective state, and the sense of belonging follows from there. The finding refines the contagion account, which has tended to treat affect as if it could leap between people unmediated; in a rehearsal the leap is staged through observable synchrony, which gives the process a behavioral substrate one can watch and code. What the design cannot show is that the sequence is irreversible, and over a season cohesion very probably feeds back into the ease of synchronizing.

Why personality conditioned the front of the chain while culture conditioned the back is the more interesting result. Extraversion and agreeableness both steepened the climb from expression to synchrony, though probably for different reasons. Extraverts appear to gather expressive energy faster, their reward sensitivity pulling them into the affect of the section; agreeable members instead smooth their behavior toward the group, trading individual deviation for fit. The two routes converge on tighter synchrony while remaining motivationally distinct, and a singer high on one trait but low on the other, the agreeable introvert flagged earlier, may reach alignment by accommodation rather than by appetite. That personality acts early makes sense, since catching the affective signal is an individual event before it is a collective one. One caution belongs here: traits were measured once, and adult rank-order consistency, though substantial, leaves room for real change (Roberts and DelVecchio, 2000), so the moderator is better read as a disposition at the time of measurement than as a fixed property of the person.

Culture, by contrast, governs interpretation more than uptake. The two regions did differ in level, shared affect running about half a scale point higher where collectivistic values predominate, and we do not wish to explain that away. What the moderation adds is a difference in rate: at any given level of shared affect, collectivistically oriented members converted it into cohesion about twice as steeply as their individualistically oriented counterparts. For the former, shared emotion read as a marker of belonging worth honoring; for the latter, the same feeling was discounted as personal rather than binding. Because the measurement model proved invariant across the two regions, because the demographic differences between them were carried as covariates, and because value orientation was measured in each singer rather than read off the sampling site, the contrast is unlikely to be an artifact of how the items functioned or of who happened to be sampled, though it cannot be fully separated from everything else that distinguishes an inland provincial choir from a metropolitan one. The pattern squares with the claim of social identity theory that cohesion hinges on whether group cues are taken as self-defining. The contribution is accordingly integrative: emotional contagion supplies the front-end mechanism, social identity the back-end conversion, and each moderator operates on the segment its parent theory explains best.

Nothing in this design speaks to physiology, yet the mechanism we describe has a plausible biological counterpart worth naming. Oxytocin is the obvious candidate. Administered intranasally, it improves synchronization with an unresponsive partner in a finger-tapping task, apparently by reducing timing variability (Gebauer et al., 2016), and in a recent pilot study listeners with high endogenous levels showed markedly larger cardiovascular, electrodermal, and respiratory swings while hearing emotionally charged music, with no such difference for soothing material (Theorell et al., 2025). Read alongside evidence that respiratory and cardiac coupling in choirs depends on musical structure (Muller and Lindenberger, 2011; Vickhoff et al., 2013; Olsson et al., 2013), this suggests a neuroendocrine route by which expressive material both draws members into alignment and makes the resulting alignment feel significant. We offer the possibility as a hypothesis for designs that combine behavioral coding with hormonal and psychophysiological measurement, not as an interpretation of the present estimates.

The practical reading should be correspondingly modest. Within rehearsal settings resembling those sampled here, a conductor who wants a more cohesive ensemble has reason to invest in coordination work, such as shared breathing, unified phrasing, and deliberate attention to entries, rather than in exhortations to feel together, since alignment preceded affect in these data. Whether such drills raise cohesion when introduced deliberately is a further claim, and only a randomized comparison of rehearsal regimes could support it. Facilitators borrowing the choral model for team building or community programs should be more cautious still: participants, task, and time frame all differ, and the cultural moderation observed here implies that the same shared warmth will convert into commitment at different rates depending on what members take group feeling to mean.

## 6 Conclusion

This study set out to explain how musical emotional expression in choral rehearsal reaches group cohesion, and it answered by building and testing a dual-moderated mediation model. The empirical work supported a serial route: expression first raises behavioral synchrony, synchrony breeds shared affect, and shared affect lifts cohesion, an ordering that fit better than its reversed and parallel rivals. Personality conditioned the opening segment, with extraversion and agreeableness steepening the move from expression into synchrony, while cultural value orientation conditioned the closing segment, collectivistic frames converting shared feeling into bonding more fully than individualistic ones. The innovation lies in separating these influences by their location in the chain rather than treating them as one undifferentiated set of moderators.

Theoretically, the model extends accounts of how affect becomes cohesion by giving the transition an observable behavioral stage and by joining emotional contagion with social identity inside a single path. It also pushes the value comparison past mean differences toward genuine moderation, showing that what members take shared feeling to mean shapes conversion rather than level. For practice, the message to choral directors, and more tentatively to team facilitators and community organizers, is that coordinated action appears to come first and felt warmth second, with both read against who is singing and within which value system.

We hold these conclusions with caution. The design separates the measurements in time but manipulates nothing, so the modeled order is supported by fit comparisons rather than established by experiment, and reciprocal influence across a rehearsal season remains entirely plausible. Personality was assessed once and treated as a stable disposition, yet rank-order consistency in adulthood is high without being perfect, and agreeableness in particular can shift over a psychosocial program (Roberts and DelVecchio, 2000), which would blur the reading of a trait moderator. Self-report supplies three of the four constructs, so shared-method variance is reduced by the temporal separation and by the marker analysis rather than removed by either. Thirty-eight choirs, finally, suffice to estimate the chain but not to estimate it precisely: the power curves of Figure 8 put the agreeableness interaction on distinctly thinner ground than the other two, and it should be treated as provisional until a larger set of ensembles is available.

The sample sets further limits. Both regions lie within one country, so the value contrast, measured rather than assumed though it is, cannot stand in for the full range of cultural variation, and it remains entangled with regional differences that we controlled for but did not eliminate. Synchrony was coded from two required pieces, and because coupling depends on texture and tempo (Vickhoff et al., 2013; Olsson et al., 2013), the estimates may not transfer to repertoire of a different kind. Future work should follow choirs across a season, add cardiac, respiratory, and hormonal indices to the behavioral coding (Theorell et al., 2025; Gebauer et al., 2016), test coordination training experimentally, and extend the value contrast across countries before the pathway is taken as settled.

## Data Availability

The results supporting the conclusions of this study are presented in the article. Further inquiries can be directed to the corresponding author.

## Glossary

IRB: Institutional Review Board
SEM: Structural Equation Modeling
MSEM: Multilevel Structural Equation Modeling
CFA: Confirmatory Factor Analysis
CR: Composite Reliability
AVE: Average Variance Extracted
ICC: Intraclass Correlation Coefficient
CFI: Comparative Fit Index
TLI: Tucker-Lewis Index
RMSEA: Root Mean Square Error of Approximation
SRMR: Standardized Root Mean Square Residual
GEQ: Group Environment Questionnaire
BFI: Big Five Inventory
SD: Standard Deviation
CI: Confidence Interval
IE: Indirect Effect
CIE: Conditional Indirect Effect
FIML: Full Information Maximum Likelihood
MLR: Robust Maximum Likelihood
AIC: Akaike Information Criterion
BIC: Bayesian Information Criterion
